# 1,25(OH)_2_D_3_ Promotes the Efficacy of CD28 Costimulation Blockade by Abatacept

**DOI:** 10.4049/jimmunol.1500306

**Published:** 2015-08-14

**Authors:** David H. Gardner, Louisa E. Jeffery, Blagoje Soskic, Zoe Briggs, Tie Zheng Hou, Karim Raza, David M. Sansom

**Affiliations:** * Medical Research Council Centre for Immune Regulation, School of Immunity and Infection, Institute of Biomedical Research, University of Birmingham, Birmingham B15 2TT, United Kingdom;; † Institute of Immunity and Transplantation, University College London and Royal Free Hospital, London NW3 2PF, United Kingdom; and; ‡ Department of Rheumatology, Sandwell and West Birmingham Hospitals National Health Service Trust, Birmingham B18 7QH, United Kingdom

## Abstract

Inhibition of the CD28:CD80/CD86 T cell costimulatory pathway has emerged as an effective strategy for the treatment of T cell–mediated inflammatory diseases. However, patient responses to CD28-ligand blockade by abatacept (CTLA-4-Ig) in conditions such as rheumatoid arthritis are variable and often suboptimal. In this study, we show that the extent to which abatacept suppresses T cell activation is influenced by the strength of TCR stimulation, with high-strength TCR stimulation being associated with relative abatacept insensitivity. Accordingly, cyclosporin A, an inhibitor of T cell stimulation via the TCR, synergized with abatacept to inhibit T cell activation. We also observed that 1,25-dihydroxyvitamin D3 enhanced the inhibition of T cell activation by abatacept, strongly inhibiting T cell activation driven by cross-linked anti-CD3, but with no effect upon anti-CD28 driven stimulation. Thus, like cyclosporin A, 1,25-dihydroxyvitamin D3 inhibits TCR-driven activation, thereby promoting abatacept sensitivity. Vitamin D3 supplementation may therefore be a useful adjunct for the treatment of conditions such as rheumatoid arthritis in combination with abatacept to promote the efficacy of treatment.

## Introduction

CD4^+^ T cell effector responses are generated following the integration of signals derived from APCs. The specificity of these responses is determined by activation of the TCR by specific peptide fragments presented by MHC II (1). In addition, costimulatory pathways deliver signals that enhance T cell activation and guide differentiation (2). The initial source of costimulation is via CD28, a T cell surface protein that is constitutively expressed by resting CD4^+^ T cells. CD28 interacts with CD80 and CD86, both of which are upregulated by APCs in response to inflammation. These interactions with CD28 reduce T cell activation thresholds (3), promote effector T cell survival (4), and enhance cytokine expression (5). CD28 costimulation is therefore widely viewed as an essential requirement for T cell activation and a control point that can be targeted therapeutically.

The extent of CD28 signaling is influenced by the expression of CTLA-4 on both regulatory T cells (T_reg_) and activated T cells. CTLA-4 binds to CD80 and CD86 with higher affinity than CD28 (6) and can therefore outcompete CD28 for ligand binding. These interactions result in the removal of CD80 and CD86 from APCs by CTLA-4 via *trans*-endocytosis (7). Consequently, whereas interactions between CD80 or CD86 and CD28 result in T cell costimulation, interactions with CTLA-4 inhibit T cell activation (8). The importance of CTLA-4 regulation is emphasized by the lethal lymphoproliferative disorder seen in CTLA-4^−/−^ mice (9, 10). This loss of immune regulation in the absence of CTLA-4 appears to be driven by CD28 because the interruption of CD28 signaling prevents disease (11).

The regulatory function of CTLA-4 has been harnessed in a clinical setting for the treatment of T cell–mediated inflammatory diseases, through the use of the CTLA-4-Ig fusion protein. The efficacy of this approach has been validated in animal models of collagen-induced arthritis, systemic lupus erythematosus, autoimmune diabetes, experimental autoimmune encephalomyelitis, and organ transplantation models (12). Several clinical trials have also demonstrated CTLA-4-Ig (abatacept) to be effective in the treatment of rheumatoid arthritis; however, a significant proportion of patients displays a limited clinical response (13).

There are several plausible explanations for a lack of efficacy of abatacept in subgroups of patients. For example, the timing of costimulation blockade may not overlap with a time frame in which T cell activation is relevant in the disease setting. Alternatively, as seen in some animal models (14, 15), T cell effector responses may not always require CD28 costimulation, and other T cell stimulatory pathways may be able to compensate for its absence. Finally, abatacept may not be effective in blocking CD28 costimulation in all settings; this view led to the development of a variant CTLA-4-Ig (belatacept) that binds to CD80/CD86 with higher affinity (16) and that has been used in renal transplantation (17). However, the higher affinity of belatacept may increase the risk of adverse events, including acute rejection, and could impact T_reg_ homeostasis (18). An alternative approach to using increased affinity analogs would be to understand the limitations of abatacept, such as conditions under which T cells become costimulation independent, to increase its potency through use in combination with other agents.

In this study, we show that the level of abatacept sensitivity is inversely associated with the strength of TCR signaling. In addition we found that 1,25-dihydroxyvitamin D3 [1,25(OH)_2_D_3_] acts directly upon T cells to inhibit their activation driven by the TCR in the absence of costimulation. Thus, by increasing reliance on CD28 costimulation, 1,25(OH)_2_D_3_ renders T cell responses more abatacept sensitive. These data suggest that vitamin D3 supplementation may be a simple approach to improve outcomes of abatacept treatment in patients with T cell–mediated inflammatory diseases.

## Materials and Methods

### Cell culture

Chinese hamster ovary (CHO) cells were cultured in DMEM (Life Technologies, Paisley, U.K.) supplemented with 10% v/v FBS (Biosera, Uckfield, U.K.), 50 U/ml penicillin and streptomycin (Life Technologies), and 200 μM l-glutamine (Life Technologies) and incubated at 37°C in a humidified atmosphere of 5% CO_2_. CHO cell lines expressing CD80, CD86, FcRγII (CD32; FcR), or FcR/CD80 were generated, as previously described (7).

### Cell isolation

PBMCs were isolated from leukocyte reduction system cones (National Blood Service, Birmingham, U.K.) by Ficoll density gradient centrifugation. CD4^+^CD25^−^ T cells were purified using an EasySep negative selection Ab mixture (StemCell Technologies), according to manufacturer’s instructions. Primary human monocytes were purified from PBMCs using an EasySep monocyte enrichment mixture (StemCell Technologies). For monocyte-derived dendritic cell (DC) differentiation, monocytes were cultured in the presence of GM-CSF (800 U/ml; Berlex Laboratories, Richmond, CA) and IL-4 (500 U/ml; Miltenyi Biotec, Bisley, U.K.) for 7 d.

### CD4^+^CD25^−^ T cell stimulation

A total of 1 × 10^5^ T cells was cocultured with either 2 × 10^4^ allogeneic DCs or glutaraldehyde-fixed CHO cells in 96-well plates for 5 d (unless otherwise stated) in RPMI 1640 supplemented with 10% FBS, 50 U/ml penicillin and streptomycin, and 200 μM l-glutamine. Stimulations were treated with anti-CD3 (OKT3; 500 ng/ml, unless otherwise stated), anti-CD28 (9.3; 500 ng/ml), or toxic shock syndrome toxin (TSST)-1 at indicated concentrations (Toxin Technology, Sarasota, FL). Alternatively, T cells were stimulated for 5 d with Dynabeads Human T-Activator CD3/CD28 beads (Life Technologies). Where indicated, stimulations were treated with abatacept (20 μg/ml; Bristol Myers Squibb), 1,25(OH)_2_D_3_ (10 nM; Sigma-Aldrich), or cyclosporin A (CsA; Sigma-Aldrich). The concentration of 1,25(OH)_2_D_3_ used was determined from previous experience (19) and similar to those used by others (20, 21). The vehicle for 1,25(OH)_2_D_3_ was ethanol, which was diluted to a final concentration of 0.01% (v/v) during experiments; vehicle controls showed no effect upon experimental outcomes. PBS vehicle was used for all other reagents.

### Flow cytometry

The following Abs were used: anti-CD25 (2A3) conjugated to FITC; anti–CTLA-4 (BNI3), anti-ICOS (DX29), anti-OX40 (ACT35), and anti-STAT5 (pY694) conjugated to PE; anti–PD-1 (MIH4), anti-CD28 (CD28.2), anti-CD62L (DREG-56), and anti-CD71 (M-A712) conjugated to allophycocyanin (all from BD Biosciences); anti-FOXP3 (PCH101) conjugated to allophycocyanin (eBioscience); and anti-TCR vβ2 conjugated to PE (Beckman Coulter Immunotech, Marseille, France). For analysis of cell surface proteins, cells were recovered following stimulation, washed once by centrifugation in PBS, and incubated with relevant Abs in PBS supplemented with 2% (v/v) goat serum (Sigma-Aldrich) for 30 min at 4°C. A FOXP3 staining buffer kit (eBioscience) was used for intracellular staining of FOXP3 and CTLA-4 in accordance with manufacturer’s instructions. Staining for pSTAT5 was performed using a BD Phosflow buffer kit (BD Biosciences). For the detection of cytokine expression, T cells were stimulated for 5 d under indicated conditions and then restimulated for 4 h with 50 ng/ml PMA (Sigma-Aldrich) and 1 μM ionomycin (Sigma-Aldrich) in the presence of 10 μg/ml brefeldin A (Sigma-Aldrich). Cells were then washed with PBS and fixed with 3% (w/v) paraformaldehyde in PBS for 12 min at room temperature. Subsequently, cells were permeabilized with 0.1% (w/v) saponin in PBS and stained at room temperature for 30 min. All data were acquired using a CyAn ADP Flow Cytometer (DakoCytomation, Ely, U.K.) and were analyzed using FlowJo software (Tree Star, Ashland, OR).

### CD4^+^ T cell proliferation assays

Prior to stimulation, CD4^+^CD25^−^ T cells were labeled with CellTrace Violet (Life Technologies). Following stimulation, T cell proliferation was analyzed by flow cytometry. Proliferation profiles were modeled using the Flowjo proliferation platform to determine the division index, which represents the average number of cell divisions undergone by a T cell in the original population.

### Detection of T cell apoptosis

Following 5-d incubation, T cell apoptosis was determined by analysis of mitochondrial depolarization, a marker of early apoptosis (22). Cells were washed and incubated with 23 ng/ml 3,3′-dihexyloxacarbocyanine iodide (DiOC_6_; Molecular Probes, Eugene, OR) for 20 min at 37°C. Cells were analyzed by flow cytometry, and apoptotic cells were characterized as a DiOC_6_^low^ population that was verified by forward/side scatter profiles.

### Statistics

Statistical analyses were performed using Prism 5.0 software (GraphPad Software, La Jolla, CA). The *p* values <0.05 were considered significant.

## Results

### Efficacy of CD28 costimulation blockade by abatacept is determined by the quality of TCR stimulation

**FIGURE 1.**
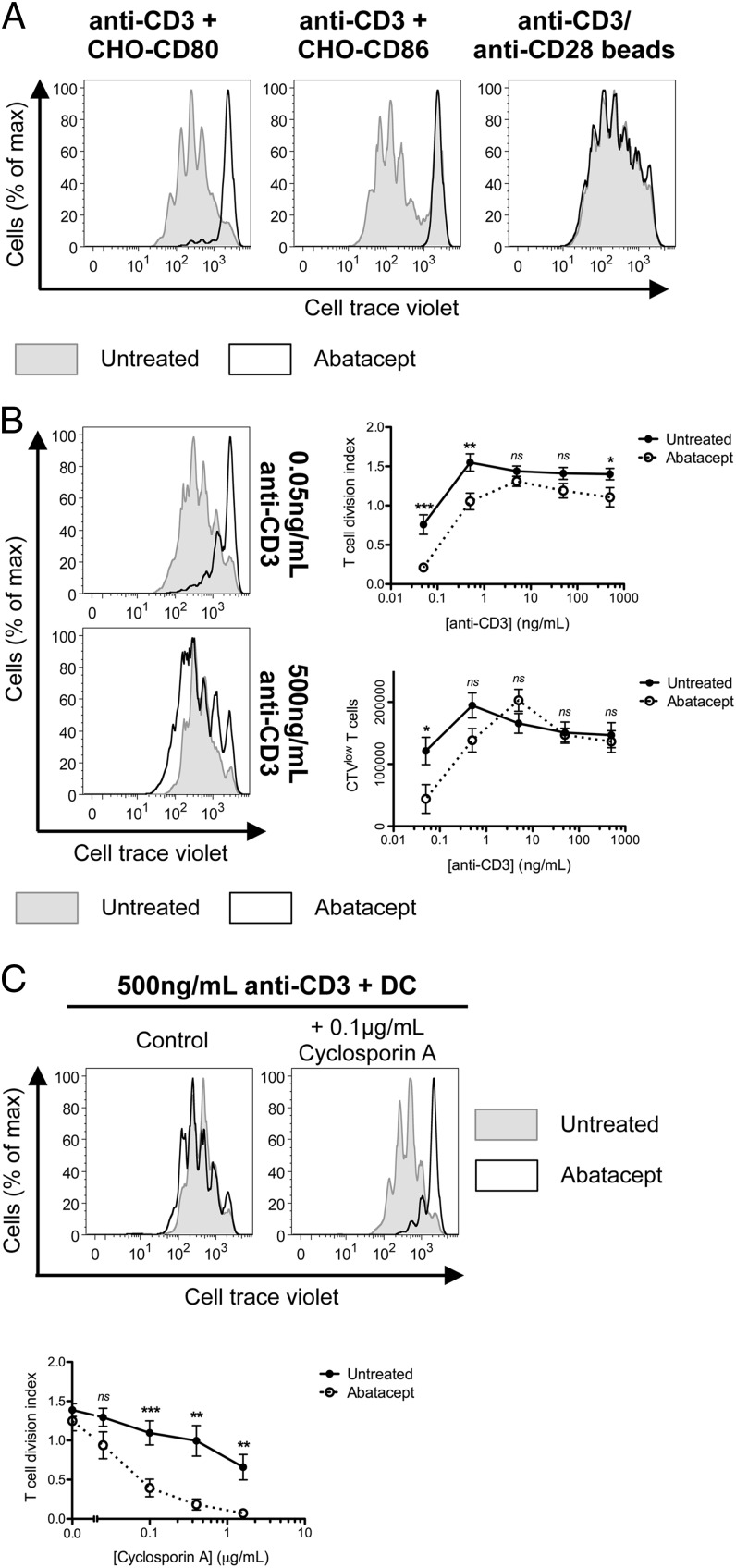
Efficacy of CD28 blockade by abatacept is dependent upon strength of TCR stimulation. (**A**) CellTrace Violet–labeled CD4^+^CD25^−^ T cells were incubated with 500 ng/ml anti-CD3 and either CHO-CD80, CHO-CD86, or anti-CD3/anti-CD28–coated beads and with or without 20 μg/ml abatacept (bold line) for 5 d, followed by flow cytometric analysis of CellTrace Violet dilution. Flow cytometry data from one representative experiment of more than five performed. (**B**) CellTrace Violet–labeled T cells were stimulated with indicated anti-CD3 concentrations and DCs for 5 d. Representative flow cytometry data and combined data from independent experiments are shown (*n* = 8) expressed as mean ± SE for division index values and total number of proliferating CellTrace Violet^low^ T cells. (**C**) CellTrace Violet–labeled T cells were stimulated with DCs and 500 ng/ml anti-CD3 for 5 d and were treated with indicated CsA concentrations, 20 μg/ml abatacept, or both. Representative flow cytometry data and mean division index ± SE values from independent experiments (*n* = 7). *p ≤ 0.05, **p ≤ 0.01, ***p ≤ 0.001, two-tailed paired *t* test. *ns*, not significant.

To test the efficacy of T cell stimulation blockade by abatacept in vitro, we stimulated CellTrace Violet–labeled CD4^+^CD25^−^ human T cells with soluble anti-CD3 and CHO cells expressing either CD80 or CD86. Treatment with a saturating abatacept concentration (20 μg/ml) robustly inhibited T cell proliferation driven by either CD80 or CD86 (Fig. 1A). As predicted, abatacept had no impact upon T cell proliferation driven by anti-CD3/anti-CD28–coated beads due to the absence of CD28 ligands in this system (Fig. 1A). These experiments therefore demonstrated the specificity and efficacy of abatacept blockade.

Surprisingly, during experiments using ligand-expressing DCs, we noted that abatacept had a limited impact on T cell proliferation when CD4^+^CD25^−^ T cells were stimulated with DCs in conjunction with the same concentrations of soluble anti-CD3 (500 ng/ml anti-CD3) that was used with CHO transfectants. However, we found significant influence of anti-CD3 concentration on the effect of abatacept treatment such that, at lower anti-CD3 concentrations (≤0.5 ng/ml anti-CD3), T cell proliferation was strongly inhibited by abatacept (Fig. 1B). This indicated that the strength of TCR signaling had a significant effect on abatacept sensitivity.

CsA is a calcineurin inhibitor that acts to prevent NFAT translocation downstream of TCR stimulation (23). Therefore, we stimulated T cells in the presence of CsA to further investigate the impact of inhibiting the TCR pathway in our experiments. Interestingly, neither CsA (0.1 μg/ml) nor abatacept (20 μg/ml) alone robustly inhibited T cell proliferation in response to high anti-CD3 concentrations (500 ng/ml). However, we observed a significant synergy between abatacept and CsA such that the effect of abatacept was enhanced by CsA treatment (Fig. 1C). Taken together, these data demonstrate that although abatacept was effective at limiting the availability of CD28 costimulation, the requirement for CD28 signaling depended on the strength of TCR engagement.

### Abatacept blockade alters the expression of effector molecules on proliferating T cells

**FIGURE 2.**
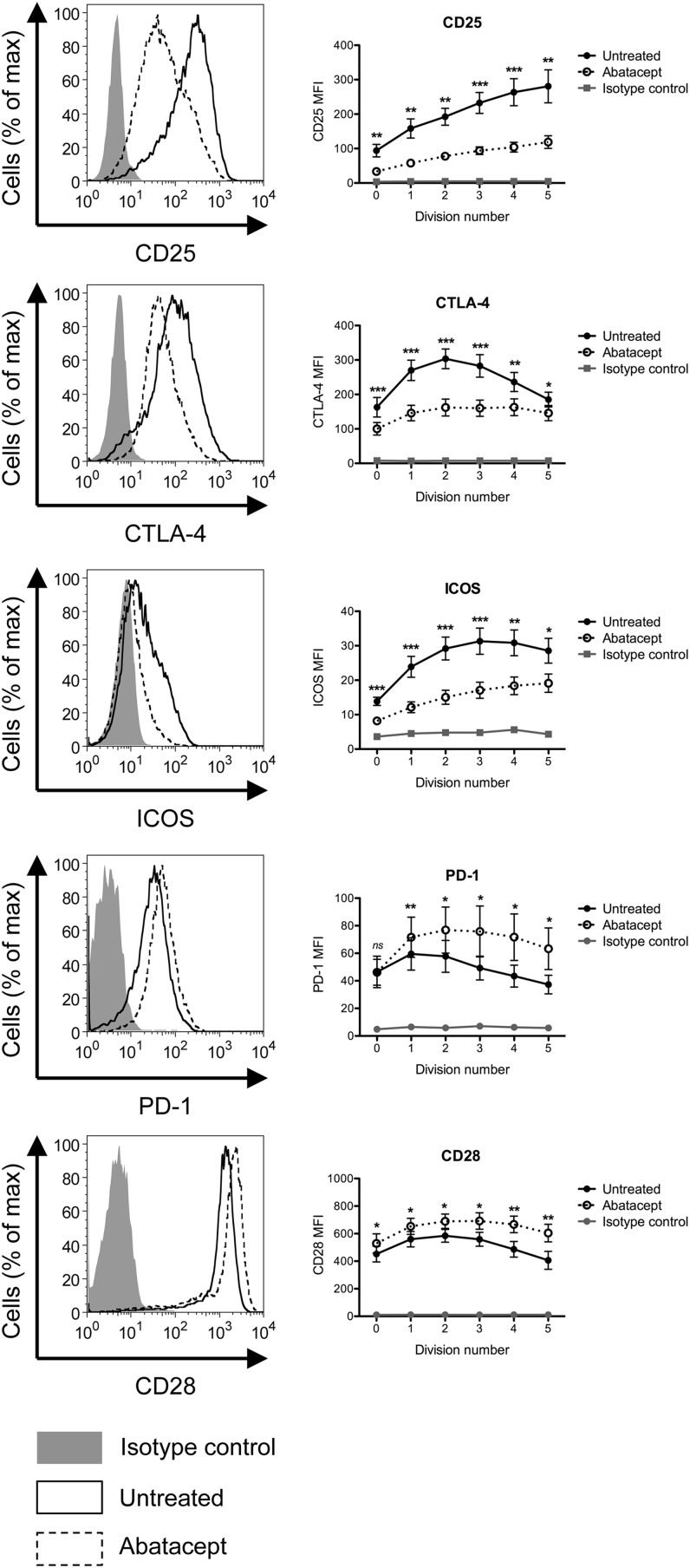
High-dose anti-CD3 promotes abatacept-resistant proliferation of T cells with an altered phenotype. Activated T cells stimulated for 5 d with DCs and 500 ng/ml anti-CD3 ± (20 μg/ml) abatacept were assessed for the activation markers indicated by flow cytometric analysis. Representative data (gated only on divided CellTrace Violet^low^ T cells) are shown as histograms and as mean fluorescence intensity (MFI) values at each CellTrace Violet division number from eight independent experiments for which data points represent mean ± SE values. *p ≤ 0.05, **p ≤ 0.01, ***p ≤ 0.001, two-tailed paired *t* test. *ns*, not significant.

Because T cell proliferation was only weakly suppressed by abatacept when stimulating with higher anti-CD3 concentrations, we wanted to determine whether the absence of costimulatory signaling still affected T cells under these conditions. We therefore assessed the expression of a variety of T cell molecules following activation either with or without abatacept treatment. Despite the fact that we only analyzed dividing cells, which by definition are activated, abatacept markedly reduced expression of the IL-2Rα chain, CD25, as well as CTLA-4 and ICOS. In contrast, abatacept prevented downregulation of PD-1 by T cells that had undergone multiple rounds of division. Additionally, we observed increased expression of CD28 itself in the presence of abatacept (Fig. 2). Therefore, although blockade of costimulation did not stop T cell proliferation, abatacept was still effective in limiting CD28 stimulation and resulted in an alteration of the phenotype of proliferating T cells.

### 1,25(OH)_2_D_3_ enhances the efficacy of abatacept

**FIGURE 3.**
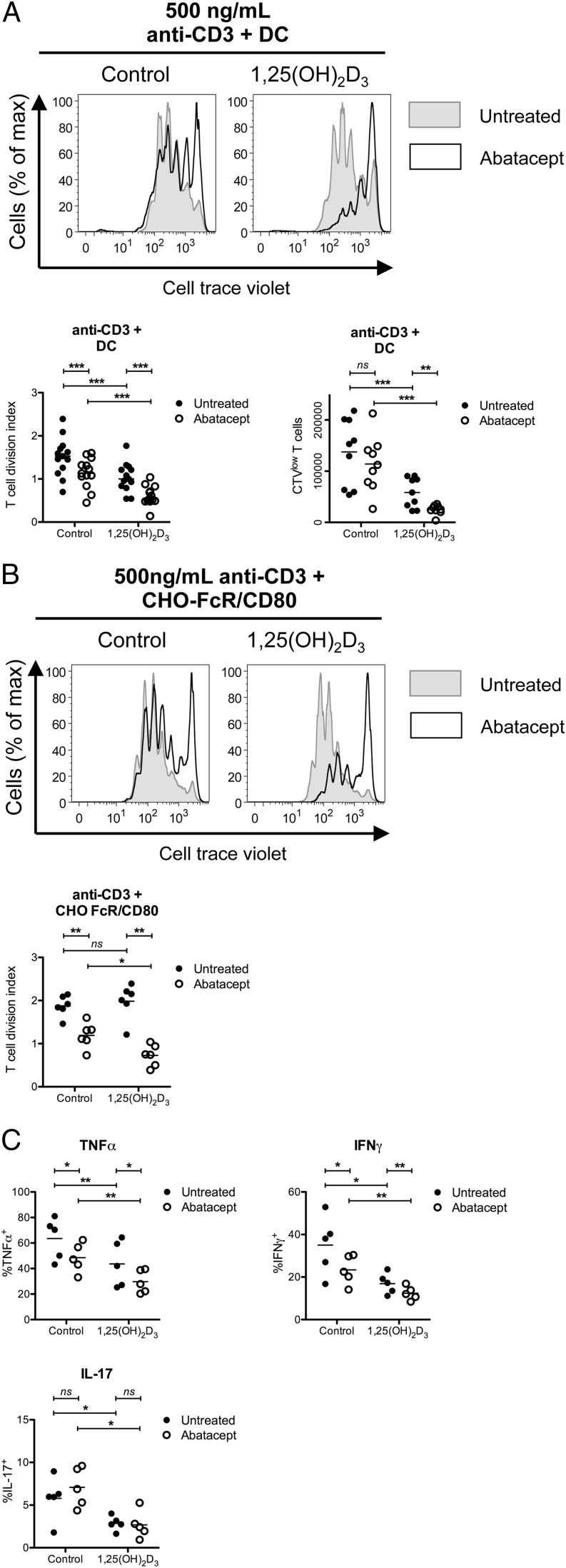
1,25(OH)_2_D_3_ increases the suppression of T cell proliferation by abatacept in a T cell–intrinsic manner. CellTrace Violet–labeled CD4^+^CD25^−^ were incubated with 500 ng/ml anti-CD3 and allogeneic DCs (**A**) or CHO-FcR/CD80 (**B**), with or without 20 μg/ml abatacept and 10 nM 1,25(OH)_2_D_3_ for 5 d. Flow cytometric data showing CellTrace Violet dilution from a representative experiment and combined data from independent experiments expressed as either division index values or total numbers of proliferating (CellTrace Violet^low^) T cells. Horizontal bars represent mean values. (**C**) T cells were stimulated with anti-CD3 and DCs for 5 d, and the expression of indicated inflammatory cytokines was assessed by flow cytometry. Data from independent experiments (*n* = 5); horizontal bars represent mean values. *p ≤ 0.05, **p ≤ 0.01, ***p ≤ 0.001, paired two-tailed *t* test. *ns*, not significant.

Previous studies have identified effects of 1,25(OH)_2_D_3_ upon CD4^+^ T cell activation and differentiation (24). We therefore wished to determine whether 1,25(OH)_2_D_3_ affected the inhibition of T cell responses by abatacept. In the presence of DCs, both abatacept and 1,25(OH)_2_D_3_ revealed independent suppressive effects on T cell proliferation. However, T cell stimulation under these conditions revealed more robust suppression of proliferation in the presence of both abatacept and 1,25(OH)_2_D_3_ (Fig. 3A). Moreover, abatacept did not obviously influence cell death, either in the presence or absence of 1,25(OH)_2_D_3_, as indicated by DiOC_6_^low^ staining cells following 5-d stimulation (Supplemental Fig. 1).

To determine whether 1,25(OH)_2_D_3_ was acting upon the DC, T cell, or both, we stimulated T cells with anti-CD3 in the presence of fixed CHO cells expressing FcR and CD80. This system provided costimulation via a fixed artificial APC that could not be influenced by 1,25(OH)_2_D_3_. Interestingly, 1,25(OH)_2_D_3_ alone failed to inhibit T cell proliferation under these conditions of stimulation (Fig. 3B) in contrast to DC-mediated activation. However, once again we observed a significant interaction between 1,25(OH)_2_D_3_ and abatacept, demonstrating that 1,25(OH)_2_D_3_ enhanced the suppression of T cell proliferation by abatacept even in the absence of live APCs (Fig. 3B). These data support a T cell–intrinsic mechanism whereby 1,25(OH)_2_D_3_ acts on T cells to enhance the control of responses by abatacept.

We also investigated the impact of 1,25(OH)_2_D_3_ and abatacept cotreatment on the expression of inflammatory cytokines in the context of T cell activation, which have previously been found to be inhibited by 1,25(OH)_2_D_3_ (Fig. 3C). Again we found independent and additive effects of abatacept and 1,25(OH)_2_D_3_ in reducing the frequencies of proliferating T cells that were IFN-γ^+^ and TNF-α^+^. The combination of both agents favored the suppression of inflammatory cytokines. Interestingly, we observed a slight trend toward increased IL-17^+^ T cells when blocking CD28 costimulation, in keeping with previous observations (25); however, this effect was lost in the presence of 1,25(OH)_2_D_3_. Together, these data suggest that 1,25(OH)_2_D_3_ supplementation enhances the efficacy of abatacept treatment affecting both T cell proliferation and inflammatory cytokine production.

### 1,25(OH)_2_D_3_ supplementation supports a CD28-driven proregulatory phenotype by activated T cells

**FIGURE 4.**
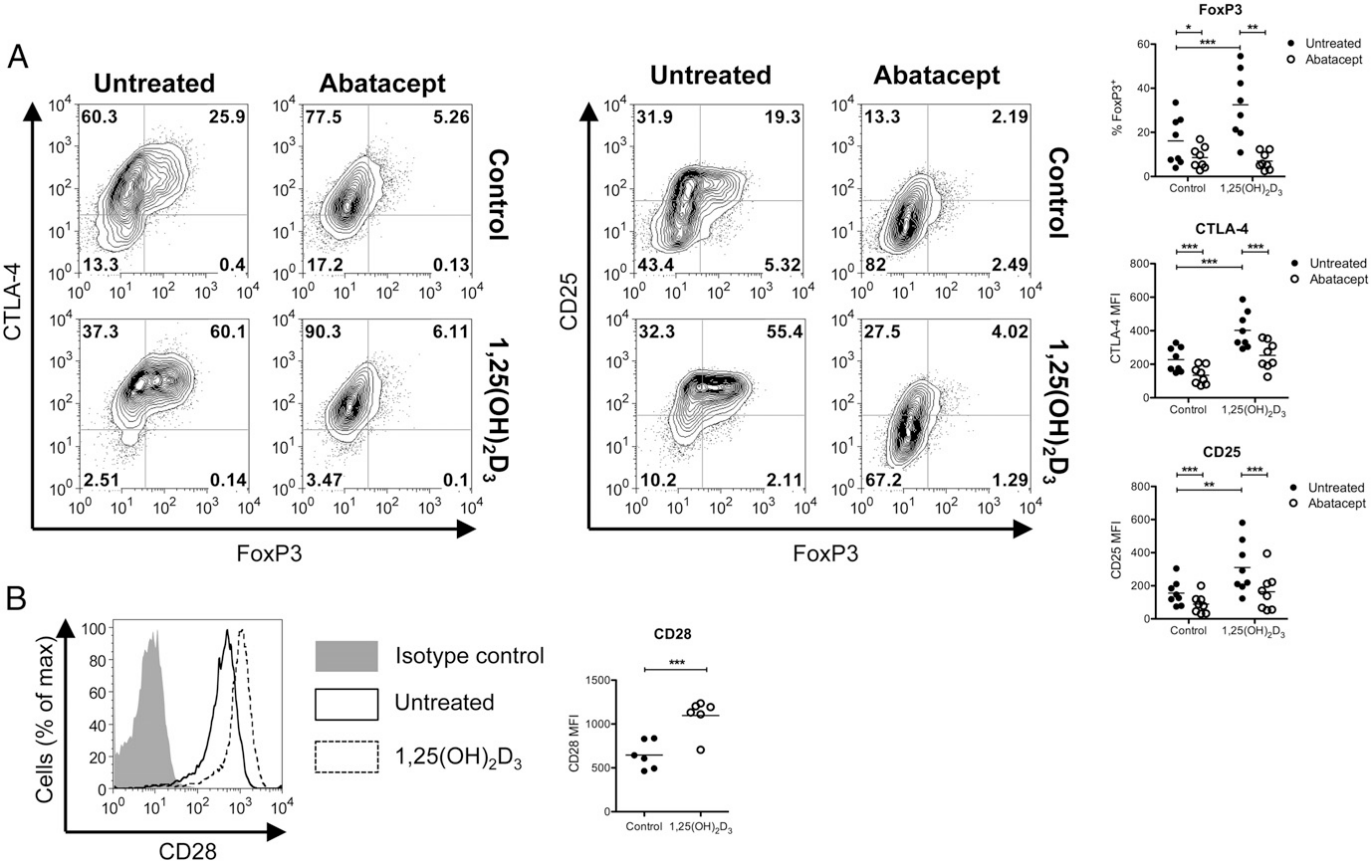
1,25(OH)_2_D_3_ supplementation promotes a T_reg_ phenotype. (**A**) T cells were stimulated with anti-CD3 and DCs in the presence of 20 μg/ml abatacept, 10 nM 1,25(OH)_2_D_3_, or both for 5 d. After 4-d stimulation, T cells were fixed, permeabilized, and stained for CD25, CTLA-4, and FOXP3. Flow cytometry data showing FOXP3 against CTLA-4 and CD25 expression and data for CD25, CTLA-4, and FOXP3 from individual experiments (*n* = 8). Horizontal bars represent mean values. (**B**) T cells stimulated in the presence or absence of 10 nM 1,25(OH)_2_D_3_ were assessed for CD28 expression after 5 d by flow cytometry. Representative flow cytometry data and combined data from independent experiments (*n* = 6). *p ≤ 0.05, **p ≤ 0.01, ***p ≤ 0.001, paired two-tailed *t* test.

To investigate further the influence of 1,25(OH)_2_D_3_ on T cell costimulatory pathways, we assessed T cell activation with particular regard to the interplay between 1,25(OH)_2_D_3_ and abatacept in the expression of markers of T cell regulation. These experiments revealed that 1,25(OH)_2_D_3_ promoted the expression of CD25 and CTLA-4 with an increased proportion of T cells expressing FOXP3 (Fig. 4A). However, the increased expression of these molecules was CD28 dependent because in the presence of abatacept this upregulation failed to occur. Interestingly, the expression of CD28 itself was significantly higher in the presence of 1,25(OH)_2_D_3_, suggesting that supplementation promotes CD28 engagement (Fig. 4B)_._ These data provide evidence that 1,25(OH)_2_D_3_ may actively increase CD28 costimulation, thereby facilitating the generation of a CD28-driven proregulatory phenotype among activated T cells.

### 1,25(OH)_2_D_3_ suppresses anti-CD3– but not anti-CD28–driven T cell activation

**FIGURE 5.**
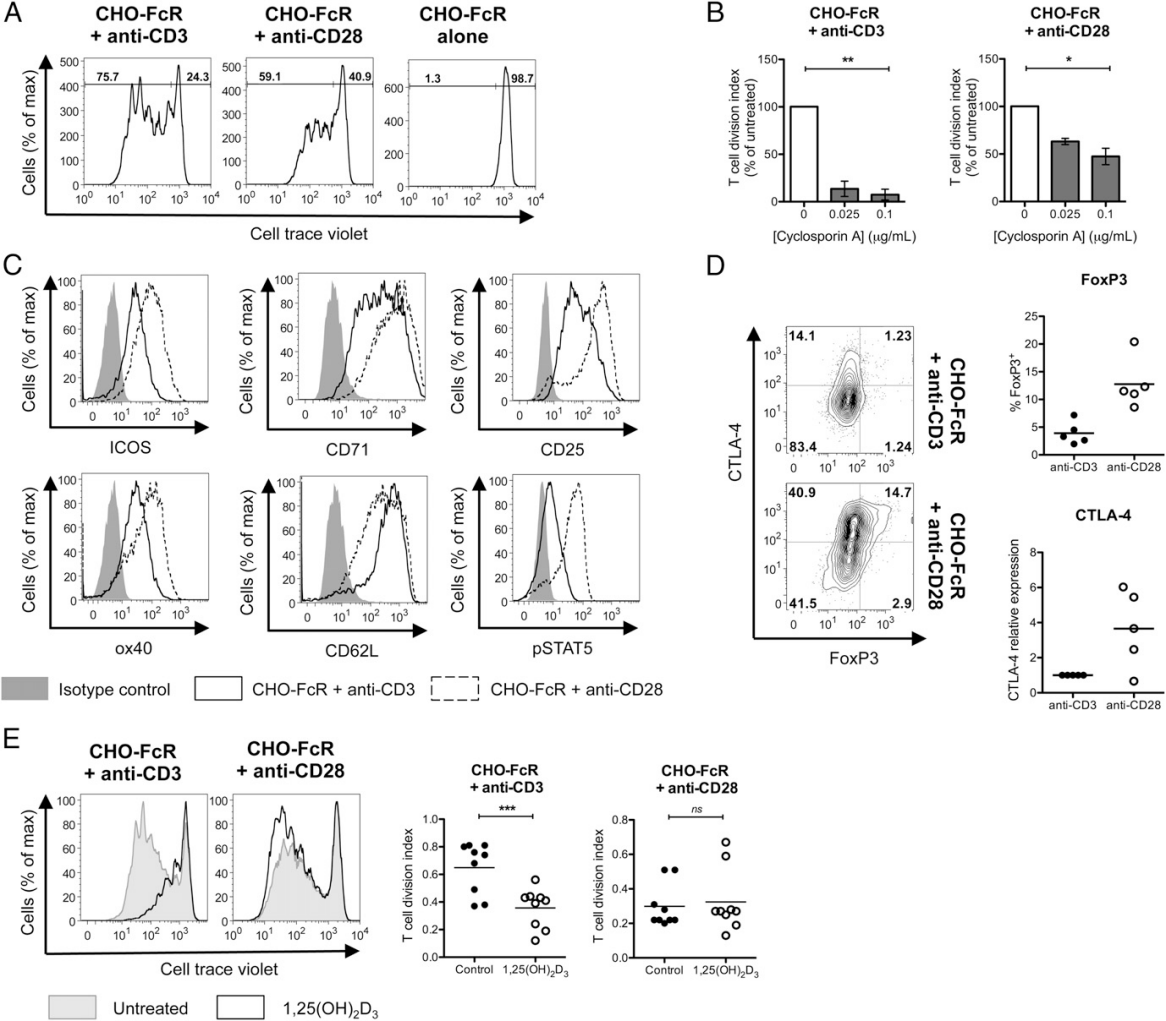
1,25(OH)_2_D_3_ suppresses TCR but not CD28-induced T cell proliferation. CellTrace Violet–labeled CD4^+^CD25^−^ T cells were stimulated with either 500 ng/ml anti-CD3 or anti-CD28 cross-linked by a FcR-expressing CHO cell (CHO-FcR). (**A**) Characteristic division at 5 d is indicated by CellTrace Violet dilution. Numbers indicate the percentage of divided (CellTrace Violet^low^) and undivided (CellTrace Violet^high^) cells. (**B**) Stimulations from (A) were treated with indicated CsA concentrations. Bar charts show the division index values at each concentration plotted as a percentage of the division index for the untreated control from independent experiments (*n* = 3). (**C**) Expression of ICOS, CD71, OX40, and CD62L by dividing T cells was measured by immunofluorescence labeling and flow cytometric analysis. Representative histograms gated on CellTrace Violet^low^ T cells from *n* > 3 experiments are shown. (**D**) CTLA-4 and FOXP3 expression following anti-CD3 or anti-CD28 treatment was determined by intracellular staining after 4 d. Representative flow cytometry data and values from independent experiments (*n* = 5). (**E**) CD4^+^CD25^−^ CellTrace Violet–labeled T cells were stimulated with anti-CD3 or anti-CD28 with or without 10 nM 1,25(OH)_2_D_3_. Representative CellTrace Violet histograms showing the impact of 10 nM 1,25(OH)_2_D_3_ on anti-CD3– and anti-CD28–driven T cell at 5 d and data from multiple independent experiments. *p ≤ 0.05, **p ≤ 0.01, ***p ≤ 0.001, paired two-tailed *t* test.

Because T cell proliferation in the presence of abatacept occurred in the context of high-intensity anti-CD3 signaling, but was rendered abatacept sensitive by 1,25(OH)_2_D_3_, we wished to further explore the hypothesis that 1,25(OH)_2_D_3_ targets the TCR/CD3 pathway, thereby making T cell proliferation more CD28 dependent. To compare the TCR/CD3 and CD28 pathways directly, we activated T cells using anti-CD3 or anti-CD28 cross-linked by FcR-transfected CHO cells. In this system, both pathways can promote T cell activation, as evidenced by proliferation at 5 d, with anti-CD28 possibly behaving in a similar manner to CD28 superagonists (Fig. 5A). Although fewer T cells committed to cell division in response to anti-CD28 compared with anti-CD3, those T cells that entered the cell cycle underwent an equivalent number of divisions after 5 d of stimulation and, interestingly, continued to proliferate for longer than anti-CD3–stimulated T cells (data not shown). We then compared the sensitivity of anti-CD3 and anti-CD28 stimulations to CsA. As expected, given the reliance of TCR signaling on the calcineurin/NFAT pathway, CsA potently inhibited proliferation of anti-CD3–treated cells, whereas anti-CD28–stimulated T cells were less affected by CsA (Fig. 5B), indicative of differences between anti-CD3 and anti-CD28 stimulation. Analysis of T cell activation markers also revealed differences between anti-CD3– and anti-CD28–stimulated T cells. Specifically, ICOS, OX40, and transferrin receptor (CD71) upregulation, and CD62L downregulation, were all more strongly induced by CD28 stimulation. In addition, CD28 stimulation was associated with increased CD25 expression and enhanced phosphorylation of STAT5 (Fig. 5C) and was accompanied by increased FOXP3 and CTLA-4 compared with anti-CD3 stimulation (Fig. 5D). Together, these data show that anti-CD3 and anti-CD28 promote distinct outcomes.

We next compared the impact of 1,25(OH)_2_D_3_ on T cell responses triggered via either the CD3 or the CD28 pathway. In this study, we observed that 1,25(OH)_2_D_3_ markedly reduced the proliferation of T cells stimulated by anti-CD3, but did not affect T cells stimulated by anti-CD28 (Fig. 5E). Taken together, these data show that 1,25(OH)_2_D_3_ treatment predominantly targets T cells stimulated via the CD3 pathway compared with the CD28 pathway.

### 1,25(OH)_2_D_3_ promotes suppression of superantigen-driven T cell activation by abatacept

**FIGURE 6.**
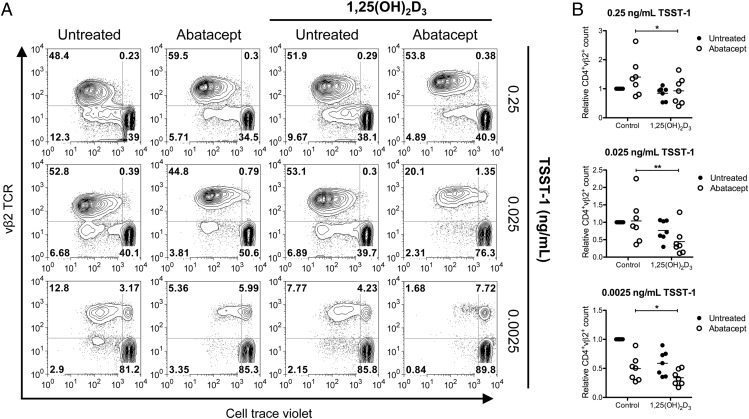
1,25(OH)_2_D_3_ promotes suppression of T cell proliferation by abatacept in superantigen stimulations. CellTrace Violet–labeled CD4^+^CD25^−^ were incubated with indicated TSST-1 concentrations and allogeneic DCs and with either 20 μg/ml abatacept, 10 nM 1,25(OH)_2_D_3_, or both for 5 d. (**A**) Flow cytometric data showing CellTrace Violet dilution and vβ2 staining from one representative experiment. (**B**) CD4^+^vβ2^+^ T cell counts were determined relative to a known quantity of beads added to each sample prior to flow cytometric analysis. Horizontal bars represent mean values from independent experiments (*n* = 7). *p ≤ 0.05, **p ≤ 0.01, paired two-tailed *t* test.

Having observed that 1,25(OH)_2_D_3_ inhibited anti-CD3–driven T cell activation, we wanted to determine its impact upon abatacept sensitivity using an alternative stimulus to anti-CD3 cross-linking. We therefore activated T cells with the staphylococcal superantigen TSST-1 that facilitates simultaneous interaction between TCRs that contain the variable β 2-domain (vβ2) and MHC II molecules (26). This allowed us to determine abatacept sensitivity in the context of a different stimulus in which TCR strength could be controlled. In this study, at high TSST-1 concentrations, we noted proliferation of both high-affinity (Vβ2^+^) T cells and low-affinity (Vβ2^−^) T cells (Fig. 6; 0.25 ng/ml TSST-1). However, the proliferation of low-affinity Vβ2^−^ T cells was more abatacept sensitive, suggesting that CD28 costimulation compensates for suboptimal TCR stimulation. In comparison, high-affinity Vβ2^+^ responder T cells remained more abatacept resistant, only becoming more susceptible to abatacept at lower TSST-1 concentrations (Fig. 6A; 0.0025 ng/ml TSST-1). Therefore, the interaction between abatacept and 1,25(OH)_2_D_3_ was more pronounced with reducing concentrations of TSST-1 (Fig. 6B). These data are therefore consistent with the concept that 1,25(OH)_2_D_3_ targets TCR-driven proliferation. Thus, using two independent systems (anti-CD3 and TSST-1), we conclude that 1,25(OH)_2_D_3_ targets TCR-driven activation, increasing CD28 dependence and thereby promoting the efficacy of abatacept.

## Discussion

As the predominant source of T cell costimulation, CD28 signaling enhances the generation of T cell effector populations via effects upon proliferation, survival, and differentiation (27). Blocking this signal using abatacept is effective for the treatment of several T cell–related diseases (12). However, responses to treatment are variable and suboptimal in a large proportion of patients. Therefore, the identification of factors that influence treatment responses along with the development of approaches to enhance responsiveness is an important clinical challenge.

The concept that TCR and CD28 cooperate toward a combined activation threshold in which CD28 provides quantitative support to the TCR signal has been previously discussed (28). This view is supported by a significant degree of overlap between TCR and CD28 signaling mediators (29–31) and a reduced TCR signaling threshold for T cell activation in the presence of costimulation (3). Furthermore, the gene expression pattern following CD28 engagement alone is comparable to that induced by TCR signaling (32–34). In this study, we found that abatacept could efficiently inhibit DC-driven T cell stimulations at low concentrations of TCR stimulus (anti-CD3 or TSST-1); however, its efficacy diminished as the dose of the TCR stimulus increased. Indeed, several studies with CD28-knockout (KO) mice suggest that T cell–mediated immune responses can occur in a CD28-deficient environment. For example, T cell responses to lymphocytic choriomeningitis virus, widely regarded as a strong antigenic stimulus, were normal in CD28-KO mice (35). Furthermore, CD28-KO T cells could mediate skin allograft rejection (36), acute lethal graft-versus-host disease (37), and diabetogenic responses in NOD mice (14). The demonstration that situations exist in which the requirement for CD28 costimulation is overcome could explain the lack of response to abatacept in some patients.

Our findings are consistent with the concept of CD28 acting as a quantitative support to the TCR signal and suggest that, under conditions of sufficient TCR stimulation, T cell responses can occur in the absence of CD28 costimulation. Conversely, sufficient CD28 costimulation may compensate for a very weak TCR signal to promote T cell activation. In this respect, we observed T cell proliferation in response to CD28 engagement in the absence of overt TCR stimulation. These responses were mediated by FcR-mediated cross-linking of anti-CD28, but did not occur in response to soluble Ab. These CD28-driven responses may occur due to the formation of tight contacts between T cell and CHO-FcR that facilitate the size-dependent exclusion of CD45 from the T cell–CHO-FcR interface as predicted by the kinetic segregation model (38). This would be analogous to T cell activation driven by CD28 superagonists (39, 40), which generate tight interfaces by binding to the membrane-proximal C”D loop of CD28 (41). Interestingly, CD28 superagonists have been found to expand T_reg_ populations in various model systems (42) consistent with our observations of a proregulatory phenotype driven by CD28 costimulation. Thus, signaling via CD28 in isolation allowed us to compare TCR- and CD28-driven signals and determine that the CD28 pathway is less sensitive to 1,25(OH_2_)D_3_.

We used a number of strategies to selectively reduce the strength of the TCR signal. The calcineurin inhibitor, CsA, has been regarded as a TCR-pathway selective inhibitor, whereas CD28 costimulation promotes CsA-insensitive T cell responses (43). When used at low concentration, CsA, like abatacept, failed to inhibit T cell proliferation; however, we found that a combination of CsA and abatacept was highly effective at inhibiting activation. Other groups have also demonstrated synergy between CsA and either anti-CD80/CD86 blocking Abs or CTLA-4-Ig for the inhibition of alloresponses in in vitro MLRs and organ transplantation models (44–47). Thus, CsA may serve to improve clinical responses to abatacept therapy. However, there are considerable adverse effects of calcineurin inhibition that limit this option (48); hence, alternative TCR-specific inhibitors are of interest. In this study, we have identified a novel role for 1,25(OH)_2_D_3_ as an inhibitor of TCR- but not CD28-driven T cell responses. Importantly, like CsA, 1,25(OH)_2_D_3_ was able to enhance the efficacy of abatacept in the blockade of anti-CD3– and DC-driven T cell stimulations.

Vitamin D has diverse immunomodulatory properties, influencing both innate and adaptive immunity. Because both APCs and T cells express the vitamin D receptor, effects of 1,25(OH)_2_D_3_ on T cell responses may occur through either a direct effect on the T cell or an indirect effect via the APC (24). By using an artificial fixed CHO system to stimulate T cells, we confirmed that 1,25(OH)_2_D_3_ can inhibit TCR-induced proliferation and promote abatacept sensitivity via a direct effect on the T cell. Our finding that 1,25(OH)_2_D_3_ can inhibit T cell proliferation is consistent with reports that have demonstrated vitamin D–mediated inhibition of cell cycle progression in various cell lineages, particularly at the G_1_/S phase transition (49). However, mixed findings are reported for the effect of vitamin D upon T cell proliferation. For example, whereas vitamin D has been found to inhibit T cell proliferation driven by PHA (50–52) in vitro, this did not occur when T cells were also costimulated with anti-CD28 (52). Our identification that 1,25(OH)_2_D_3_ acts primarily on TCR-mediated activation could explain the discrepancy, wherein, the greater the TCR dependency of the stimulation, the greater the effect of 1,25(OH)_2_D_3_.

In addition to influencing T cell activation, vitamin D has been widely reported to modify T cell effector function, for example, by inhibiting proinflammatory cytokine production by Th1, Th9, and Th17 cells (21, 53–56) and biasing differentiation toward Th2 responses (57). Notably, vitamin D also promotes a regulatory phenotype characterized by increased expression of FOXP3 and CTLA-4 (55). Interestingly, Th1 (58)- and Th17 (25)-type responses are favored by TCR signaling, whereas CD28 signaling favors Th2 (58) and T_reg_ (59). Consistent with these findings, we observed that abatacept blocked the induction of regulatory markers, including FOXP3 and CTLA-4, even under conditions in which it was not effective at suppressing proliferation. This highlights the inherent tension between inhibiting effector responses and at the same time influencing regulatory outcomes (60). The fact that vitamin D promotes a regulatory outcome and suppresses inflammatory cytokine production favors its use in combination therapy; nonetheless, its ability to induce regulatory markers on activated T cells was still reduced by abatacept. The fact that the two agents combined to more potently inhibit T cell proliferation is likely to be beneficial. Importantly, 1,25(OH)_2_D_3_ continued to suppress inflammatory cytokine expression even in the presence of abatacept. Thus, greater inhibition of T cell proliferation as well as the reduction of inflammatory cytokines due to the presence of 1,25(OH)_2_D_3_ favors its combination with abatacept as a potential treatment of inflammatory diseases.

Overall, our data suggest that suboptimal responses to abatacept treatment could be associated with the strength of the TCR stimulus that drives the T cell response. Factors that may influence the strength of the TCR signal between different disease-affected individuals include autoimmunity-associated genetic variations (for example, PTPN22, a protein tyrosine phosphatase that regulates TCR signal transduction) (61) and the antigenic trigger(s). To enhance responses to abatacept in those in whom pathology is associated with a strong TCR stimulus, the TCR pathway could be targeted in addition to the CD28 stimulatory pathway. Our data demonstrate that, in cases in which abatacept-resistant T cell activation occurs due to strong TCR stimulus strength, vitamin D3 supplementation can enhance the efficacy of treatment by inhibiting TCR-driven T cell proliferation.

## Supplementary Material

Data Supplement
