# Supplementary material for: 1,25(OH)2D3 Promotes the Efficacy of CD28 Costimulation Blockade by Abatacept
Source: J Immunol. 2015 Aug 14;195(6):2657–65. doi: 10.4049/jimmunol.1500306 (PMC4560489; doi:10.4049/jimmunol.1500306)
Supplement: Data Supplement [file JI_1500306.zip › JI_1500306_Supplemental_Figure_1.pdf]

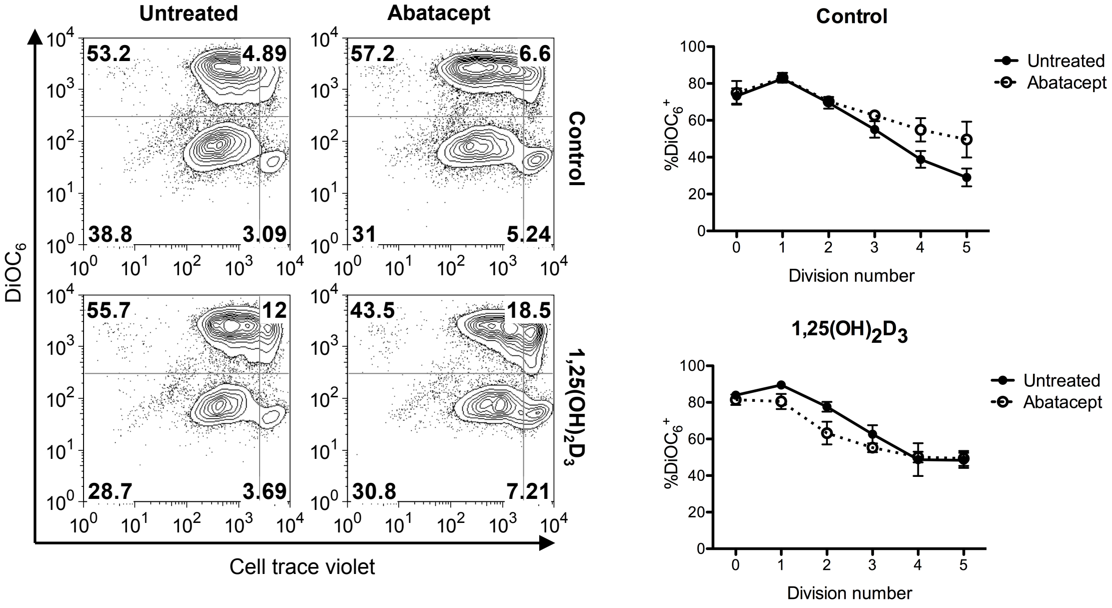

Supplemental fig. 1 Cell trace violet labeled $CD4^+$ $CD25^-$ were activated with 0.5µg/mL anti-CD3 in association with 20µg/mL abatacept and 10nM $1,25(OH)_2D_3$ for 5 days. Activated T cells were stained with $DiOC_6$ and analysed by flow cytometry with $DiOC_6^{high}$ cells representing live cells. Representative flow cytometry data and mean ±S.E values values from independent experiments (*n*=3).
